# Limitations and use of the Morpheus-V5 dual reporter virus in assessing interventions that target HIV latency

**DOI:** 10.1016/j.jviromet.2025.115236

**Published:** 2025-08-05

**Authors:** Kiho Tanaka, Youry Kim, Jesslyn Ong, Carolin Tumpach, Ajantha Rhodes, Hannah AD King, Anna C. Hearps, Michael Roche, Sharon R. Lewin

**Affiliations:** a Department of Infectious Diseases, The University of Melbourne at the Peter Doherty Institute for Infection and Immunity, Melbourne, Australia; b Burnet Institute, Melbourne, Victoria, Australia; c Infectious and Inflammatory Diseases Theme, School of Health and Biomedical Sciences, RMIT University, Melbourne, Australia; d Department of Infectious Diseases, Alfred Hospital and Monash University, Melbourne, Australia; e Victorian Infectious Diseases Service, Royal Melbourne Hospital at the Peter Doherty Institute for Infection and Immunity, Melbourne, Australia

**Keywords:** HIV, Dual-reporter, MDM, CD4 +T-cell, Latency, Latency reversal, Apoptosis

## Abstract

HIV can persist indefinitely in latently infected CD4 + T-cells as an integrated provirus with limited or no viral transcription and expression of viral proteins. We further characterised a recently described dual reporter virus, Morpheus-V5, that expresses murine heat-stable antigen and mCherry in productively infected cells (which is HIV LTR dependent) and V5 and Nerve growth factor receptor (NGFR) in latently infected cells (which is HIV LTR independent). We demonstrated successful infection of resting and activated CD4 + T-cells using Morpheus-V5 pseudotyped with either X4, R5 or dual tropic envelope proteins. We also showed that expression of NGFR (a transmembrane protein) enriched for infected cells (that contained HIV DNA) with inducible virus, however uninfected cells also expressed NGFR as a result of NGFR incorporation into virion preparations. Following treatment of CD4 + T-cells infected with Morpheus-V5 with latency reversal agents, we demonstrated an increase in the percentage of cells expressing mCherry and a decrease in the percentage of cells expressing NGFR. In addition, using this model, we showed that latent and productively infected cells had different levels of sensitivity to pro-apoptotic compounds. The Morpheus-V5 dual reporter virus has some limitations and overestimates the number of latently infected cells, but is a useful tool to investigate interventions that disrupt HIV latency.

## 1. Introduction

HIV infection of CD4 + T-cells results in either productive or latent infection, where virus integrates into the host genome but there is no or limited RNA transcription or expression of viral proteins (reviewed in (Mbonye and Karn, 2024)). Latently infected cells persist indefinitely on antiretroviral therapy (ART) and therefore are a major barrier to achieving a cure for HIV (Finzi et al., 1997; Chun et al., 1997; Wong et al., 1997). There are currently no established biomarkers to identify latently infected cells (reviewed in (Darcis et al., 2019)) and these cells persist in people with HIV on ART at extremely low frequency (Finzi et al., 1997; Ho et al., 2013). Hence, there is high interest in developing in vitro models that can fully characterise and understand features of HIV latency using primary CD4 + T-cells rather than immortalised cell lines and productively infected cells (reviewed in (Angamuthu et al., 2023)).

To better understand latent infection *in vitro*, we and others have evaluated dual reporter viruses that express a reporter protein in latently infected cells and another reporter protein in productively infected cells (Calvanese et al., 2013; Dahabieh et al., 2013; Chavez et al., 2015; Battivelli et al., 2018; Kim et al., 2019; Cai et al., 2021; Kim et al., 2022). In most of these reports, the productive reporter protein expression was regulated by the HIV long-terminal repeat (LTR) promoter, whilst the latent reporter protein expression was dependent on an LTR-independent promoter. Some HIV reporter viruses have incorporated promoters from other viruses, such as the cytomegalovirus (CMV) (Dahabieh et al., 2013) promoter and the spleen Focus Forming Virus (SFFV) (Chavez et al., 2015) promoter or an eukaryotic promoter such as elongation factor 1α (EF1α) (Calvanese et al., 2013; Chavez et al., 2015; Battivelli et al., 2018; Kim et al., 2019; Cai et al., 2021).

One of the common limitations of many dual reporter viruses has been the low frequency of latent infection, making it difficult to further characterise these cells (Calvanese et al., 2013; Chavez et al., 2015; Battivelli et al., 2018; Kim et al., 2019). This has been explained by LTR-driven transcription recruiting transcription factors preferentially and therefore suppressing the expression of the latent marker (Kim et al., 2019; Ben-Dor et al., 2006; Konopka et al., 2000). In addition, some reporter viruses still require T-cell activation prior to infection, due to low efficiency of expression of the latent reporter protein in resting T cells (Battivelli et al., 2018; Cai et al., 2021), as we have previously reported when using a dual reporter virus to directly infect resting cells as a model for pre-activation latency (Kim et al., 2019).

Morpheus-V5 is a dual reporter virus that expresses murine heat-stable antigen (HSA) and mCherry in productively infected cells (which is HIV LTR dependent) and V5 and Nerve growth factor receptor (NGFR) in latently infected cells (which is HIV LTR independent) (Kim et al., 2022). Expression of the LTR independent reporter to identify latently infected cells is regulated by the phosphoglycerate kinase (PGK) promoter, which is highly expressed in activated T cells following len-tiviral transduction (Jones et al., 2009) and is not silenced by the human silencing hub (HUSH) complex (Kim et al., 2022; Tchasovnikarova et al., 2015). The Morpheus-V5 virus encodes all viral proteins except for *Env* and can establish infection in resting CD4 + T-cells (Kim et al., 2022). Compared to other dual reporter viruses, the Morpheus-V5 system established remarkably high levels of latent infection (Kim et al., 2022) and therefore could be a valuable in vitro tool to assess cure interventions that target latency.

In this study, we aimed to assess the effects of pseudotyping viral envelope proteins with different tropism in the Morpheus-V5 backbone on the frequency of latent and activated infected T-cells, as well as monocytes-derived macrophages. We then used the reporter virus to investigate the effects of latency reversal agents (LRAs) and pro-apoptotic drugs on virus expression and death of productively and latently infected cells.

## 2. Methods

### 2.1. Ethics statement

Use of buffy coats from healthy donors (Australian Red Cross Life-blood) was approved through the University of Melbourne Office of Research Ethics and Integrity (13194).

### 2.2. Human CD4 + T-cell isolation and cell culture

Peripheral blood mononuclear cells (PBMCs) were collected from buffy coats using Ficoll-Plaque density gradient centrifugation. CD4 + T-cells were isolated using EasySep Human CD4 + T-cell Isolation Kit (StemCell Technologies, Vancouver, Canada). Isolated CD4 + T-cells were cultured at 37 degrees, 5 % CO_2_ in RPMI-1640 medium (Thermo Fisher Scientific, Carlsbad, CA) supplemented with 10 % foetal bovine serum (FBS; Cellsera, Australia), 2 mM L-glutamine, 100U/ml penicillin, 100ug/ml streptomycin (all Thermo Fisher Scientific) and 10U/ml re-combinant human interleukin-2 (IL-2) (Sigma-Aldrich, St. Louis, MO) (RF10).

### 2.3. Morpheus-V5 virus stock production

The Morpheus-V5 system including reporter genes and plasmids have been previously described (Kim et al., 2022). Briefly, 7 × 10^6^ human embryonic kidney (HEK) HEK293T cells (NIH AIDS reagent program) were transfected with 16 ug of pLAI2-V5-NGFR-HSA-mCherry-IRES-Nef and 1 ug of pSVIII Env (NIH AIDS reagent program) (Gao et al., 1996) using polyethylenimine (PEI) transfection reagent (Polysciences Asia, Warrington, PA). Env strains included X4 strains (NL4.3 and HXB2); R5 strains (YU2 and JRCSF); and dual tropic strains (89.5 and HT593.1). After 24 h, media was changed, and the supernatants were collected and filtered (0.45um) at 48 h and 72 h after transfection. The collected supernatants were concentrated using Lenti-X concentrator and resuspended in 200ul of RF10 (Takara, San Jose, CA).

### 2.4. Infection of primary CD4 + T-cells with Morpheus-V5

Isolated CD4 + T-cells were treated with/without 10 ug phytohemagglutinin (PHA) (Thermo Fisher Scientific) and cultured in 10U/ml IL-2 for 2 days before infection. Cells were infected with tissue culture infectious dose (TCID)50 unit per cell of 0.005 by spinoculation at 1200 g for 99 min. After spinoculation, cells were cultured in the presence of 10U/ml IL-2 for 2-or 5-days post infection. For experiments with LRAs, cells were cultured in the presence of 10U/ml IL-2 for 3 days and cultured in the presence of a latency reversal agent for additional 48 h. LRAs tested included JQ1 (1uM; Selleck Chemicals), PEP005 (50 nM; Tocris In Vitro Technologies), romidepsin (20 nM; Selleck Chemicals), 16 nM Phorbol 12-myristate 13-acetate (PMA) (Sigma-Aldrich) and 0.5uM ionomycin (Sigma-Aldrich) and 0.1 % DMSO. For experiments with antiretrovirals, T20 (20ug/ml; NIH AIDS reagent program) or ral-tegravir (1uM; Selleck Chemicals) was added at the time of infection and cultured in the presence of 10U/ml IL-2 for 5 days.

### 2.5. Infection of monocyte-derived macrophages with Morpheus-V5

Monocytes were matured into macrophages via plastic adherence. In brief, PBMCs were cultured in Iscove’s Modified Dulbecco’s Medium (IMDM) (Thermo Fisher Scientific) supplemented with 10 % foetal bovine serum (FBS; Cellsera, Australia), 2 mM L-glutamine, 100U/ml penicillin, 100ug/ml streptomycin (all Thermo Fisher Scientific) and 10 ng/ml macrophage colony-stimulating factor (MCSF) (Stemcell Technologies) in a 6 well plate. After 2 h, cells were washed with media and cultured with fresh media for 8 days. On day 9, cells were washed with media and virus was added directly onto cells. Cells were infected with 2.5 × 10^3^ TCID50 units’ equivalence. Cells were cultured for further 5 days in fresh media. Cells were detached from the well for FACS analysis using StemPro accutase cell dissociation reagent (Thermo Fisher Scientific) according to the manufacturer’s instructions.

### 2.6. Flow cytometry

The infected cells were stained with the LIVE/DEAD Fixable Dead Cell Stain Kit (Thermo Fisher Scientific) and anti-NGFR-Alexa Fluor 647 conjugated antibody (BD Bioscience, Franklin Lakes, NJ; C40–1457) in FACS buffer (phosphate-buffered saline (PBS) with 1 % FBS and 1 mM ethylenediaminetetraacetic acid (EDTA)). For annexin V staining, annexin V-BUV395 (BD Bioscience) was used in 1xAnnexin V binding buffer (Biolegend, San Diego, CA). Data were analysed using Omiq software from Dotmatics (Dotmatics, Boston, MA). For experiments involving isolated cell populations, fractions of cells (mCherry-NGFR-, mCherry+NGFR+, mCherry-NGFR+) were sorted from Morpheus-V5 infected CD4 + T-cells using a BD FACSAria Fusion (BD Bioscience, Franklin Lakes NJ)

### 2.7. Quantification of total HIV-1 DNA

For experiments involving quantification of total HIV-1 DNA, Morpheus-V5 virus stocks were pretreated with 10U/ml DNase (Qiagen, Hilden, Germany) for 1 h at room temperature before being added to cells. Total HIV DNA was quantified as previously described (Lewin et al., 2008). HIV copies were normalised to cell input via quantification of the host gene CCR5 (Hooker et al., 2012).

### 2.8. Western blotting

Cells were lysed in lysis buffer (Thermo Fisher Scientific) supplemented with Halt Protease inhibitor (Thermo Fisher Scientific) and supernatant was collected by centrifuging the cell lysate at 15,000 rpm for 20 min at 4 degrees. Proteins were separated by SDS-PAGE gel, transferred to polyvinylidene fluoride (PVDF) membranes and detected using anti-HIV p24 antibody (Abcam, #ab63913), anti-NGFR (p75NTR (D4B3) XP) Rabbit mAb (Cell Signaling Technology, #8238) and anti-vinculin (E1E9V) XP Rabbit mAb (Cell Signaling Technology, #13901), followed by anti-rabbit peroxidase-conjugated secondary antibody (Cell Signaling Technology, #7074).

### 2.9. Data and statistical analysis

Statistical analyses were performed using R (v4.3) and RStudio (v2023.06.1 +524). A paired *t*-test was used to compare two groups and p-values less than 0.05 were considered statistically significant.

## 3. Results

### 3.1. Pseudotyping Morpheus-V5 with diverse envelopes results in efficient latent infection

**Fig. 1.**
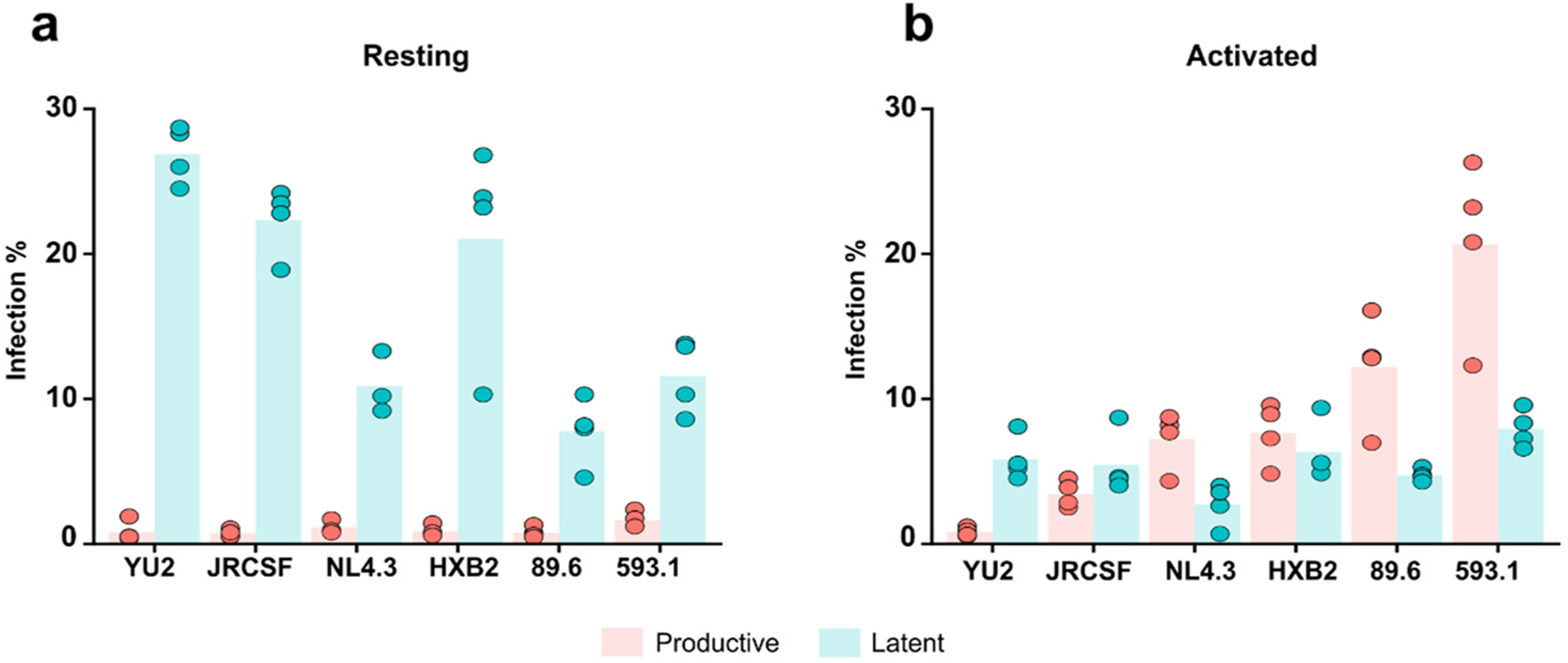
Infection with Morpheus-V5 can be established in activated and resting CD4 + T-cells using envelopes with different tropism. Resting or phytohemagglutinin (PHA) activated CD4 + T-cells were infected with Morpheus-V5 virus at tissue culture infectivity dose (TCID) 50 per cell of 0.005. CCR5 tropic (YU2, JCRSF), CXCR4 tropic (NL4.3, HXB2) and dual tropic (83.1, HT593.1) envelopes were co-transfected with the plasmid (p)Morpheus-V5 to generate reporter viruses with different tropism. Cells were harvested five days post infection. (a) The percentage of cells with productive (NGFR+mCherry+, red) and latent (NGFR+mCherry−, blue) infection are shown. The symbols represent individual donors and the height of the column the mean of n = 3–4 individual donors.

We first assessed the effects of pseudotyping Morpheus-V5 with diverse HIV *Env*. The backbone plasmid was pseudotyped with R5 (YU2, JRCSF), X4 (NL4.3, HXB2) and dual tropic (89.3, 593.1) envelopes and confirmed that both productive and latent infection could be established in resting and activated human CD4 + T-cells (Fig. 1). We found that infection of resting CD4 + T-cells led to high levels of NGFR+mCherry-(latent) cells (range 7.76 %–26.88 %) and NGFR+mCherry+ (productive) cells (range 0.73 %-1.64 %) (Fig. 1a). In activated CD4 + T-cells, the frequency of NGFR+mCherry-(latent) cells was 2.72 %-7.93 % and NGFR+mCherry+ (productive) cells was 0.83 %–20.65 %, Fig. 1b). Pseudotyping with the macrophage-tropic *Env* YU2, also resulted in latent and productive infection in monocyte-derived macrophage (MDM) (Fig S1). In summary, we demonstrated that using the Morpheus virus with multiple different envelopes, higher levels of latent infection can be established in resting compared to activated cells and that latent infection could be established with R4, X4 and dual tropic envelope proteins.

### 3.2. Expression of NGFR protein over estimates the frequency of latently infected cells with inducible virus

**Fig. 2.**
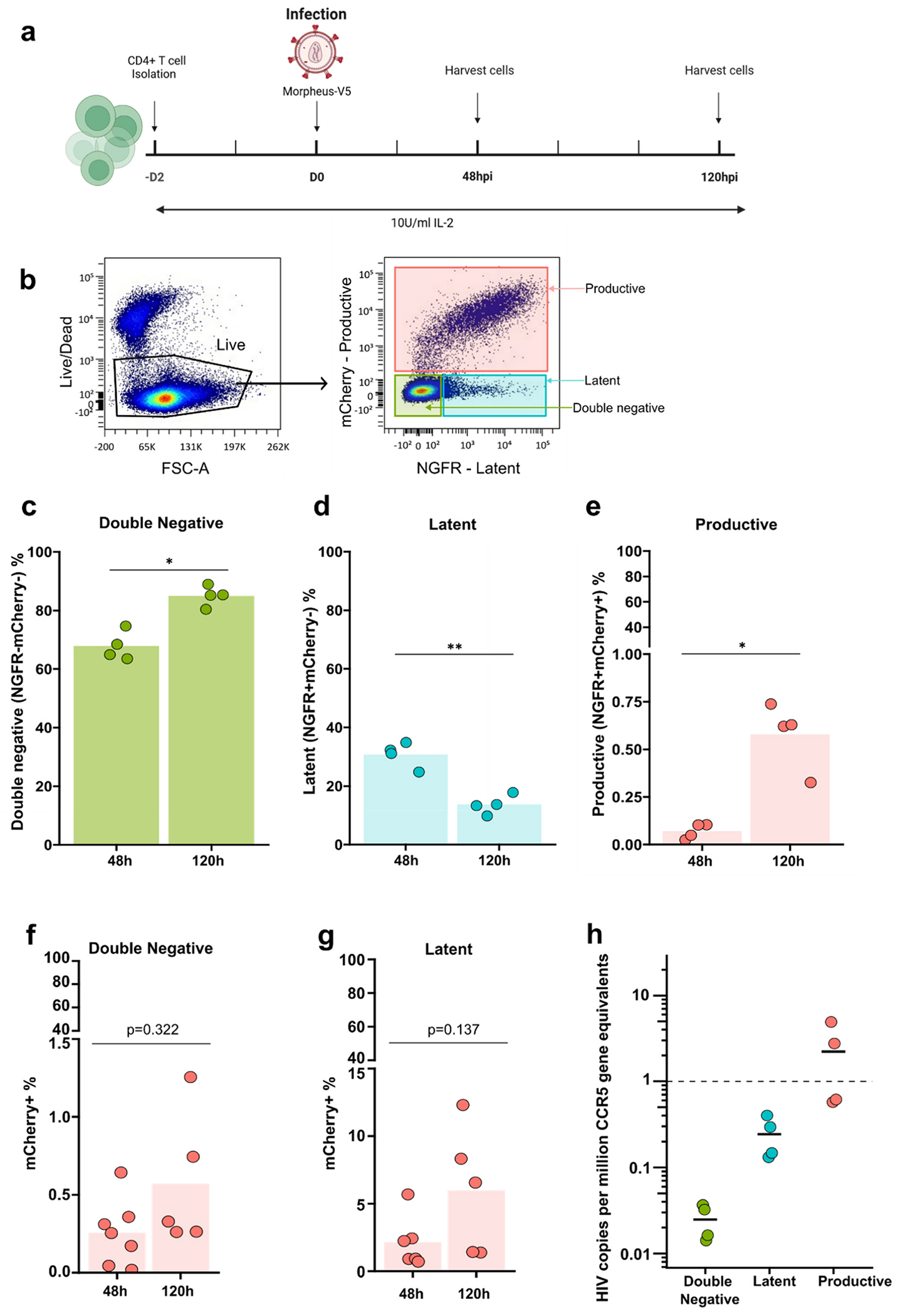
NGFR expression over estimates the number of latently infected cells. Resting CD4 + T-cells were infected with Morpheus-V5 virus at tissue culture infectivity dose (TCID) 50 per cell of 0.005. Cells were harvested at 48 h and 120 h post-infection. (a) A schematic representation of the experimental design and (b) gating strategy used to quantify infected cells. The percentage of (c) double negative (uninfected, green) cells, (d) NGFR+mCherry− (latent, blue) cells and (e) NGFR+mCherry+ (productive, red) cells as a proportion of live cells at 48 hpi and 120hpi is shown. The symbols represent individual donors and the height of the column the mean of n = 4 individual donors. After infection, cells were sorted into NGFR+ (latent) and double negative (uninfected) cells at 48 hpi and 120 hpi, followed by stimulation with16nM PMA and 0.5uM ionomycin for a further 48 h. The expression of mCherry was quantified by flow cytometry in (f) double negative (uninfected) and (g) NGFR+mCherry− (latent) cells after 48 h and 120 h. The symbols represent individual donors and the height of the column the mean of n = 5–7 individual donors. To determine the proportion of cells that contained HIV DNA, CD4 + T-cells were activated with 10ug/ml PHA (phytohemagglutinin) for 48 h and infected with DNase pretreated Morpheus-V5 at TCID 50 per cell of 0.005. Five days post-infection, cells were sorted into double negative (uninfected, green), NGFR+mCherry− (latent, blue) and NGFR+mCherry+ (productive, red) cells. The cells were then lysed, DNA extracted and real time PCR was performed to quantify (h) HIV DNA copies relative to cell number. Line represents mean of n = 4 donors. Statistical significance was calculated using a paired *t*-test. *p < 0.05; **p < 0.01.

Given the unexpectedly high frequency of NGFR+mCherry-(latent) cells in resting cells (range 7.76 %–26.88 %, Fig. 1a), we hypothesised that expression of NGFR marker may have occurred in a population of cells that did not contain latent provirus. To investigate the reasons for this, we first measured the levels of NGFR+mCherry+ (productive) and NGFR+mCherry-(latent) infection over time up to 120 h post-infection (hpi) in the absence of any intervention (Fig. 2a, b). We observed an increase in the double negative (uninfected) population over time (1.25 fold increase from mean±SEM=67.90 ± 2.50 % to 84.95 ± 1.74 %, 48 h vs 120 h, p = 0.016, Fig. 2c), a decrease in the NGFR+mCherry- (latent) population (0.45-fold reduction from mean±SEM=30.75 ± 2.11 % to 13.73 ± 1.68 %, 48h vs 120 h, p = 0.009, Fig. 2d) and a small but significant increase in the NGFR+mCherry+ (productive) population (8.24-fold increase from mean±SEM=0.07 ± 0.02 % to 0.58 ± 0.09 %, 48 h vs 120 h, p = 0.013, Fig. 2e). When cells were cultured up to 14 days post infection (dpi), we observed a further reduction in the NGFR+mCherry- (latent) population with a frequency of NGFR+mCherry- (latent) cells of 2.75 ± 1.15 % (Fig S2).

To determine if the decrease in NGFR+mCherry- (latent) cells was due to silencing of the latent promoter resulting in reduced expression of NGFR, we sorted NGFR+mCherry- (latent) and double negative (uninfected) cells at 48hpi and 120hpi and then activated the cells with PMA/ionomycin (in the absence of raltegravir since Morpheus-V5 is a single round virus) to quantify the number of cells with inducible virus. In the double negative (uninfected) cells, there were few cells with inducible virus following PMA/ionomycin stimulation (<1 %) and no change over time in culture (Fig. 2f). In the NGFR+mCherry- (latent) cells, there were cells with inducible virus, which increased between 48 and 120 hpi, however the frequency was very low and the difference did not reach statistical significance (2.14 ± 0.77 % and 5.99 ± 2.10 % mCherry+, respectively, p = 0.137, Fig. 2g). These data demonstrate that NFGR+mCherry- (latent) cells were indeed modestly enriched for cells with inducible virus, however, the frequency of these cells was low.

Finally, given prior reports that not all latent virus can be induced with a single stimulation of PMA/ionomycin (Hosmane et al., 2017) and some latent virus will be non-inducible (Ho et al., 2013), we determined the proportion of NGFR+mCherry- (latent) cells that were infected by quantifying the number of HIV copies per cell in each population at 120hpi. If all cells expressing the reporter proteins were infected, then the HIV DNA copy number per cell should be at least one. We found that the NGFR+mCherry+ (productive) cells had > 1 (mean HIV copy/cell ±SEM=2.22 ± 1.03, Fig. 2h), while the NGFR+mCherry- (latent) cells had a ratio < 1 (mean HIV copy/cell±SEM=0.20 ± 0.06, Fig. 2h) indicating that not all cells were infected. As expected, most double negative (uninfected) cells had no detectable HIV DNA (mean HIV copy/cell±SEM=0.03 ± 0.00). Taken together, we showed that expression of NGFR enriches for HIV DNA but overestimates the number of NGFR+mCherry- (latent) cells.

### 3.3. NGFR protein in virion preparations can contribute to the number of NGFR+mCherry- (latent) cells following infection of CD4 + T-cells with Morpheus-V5

**Fig. 3.**
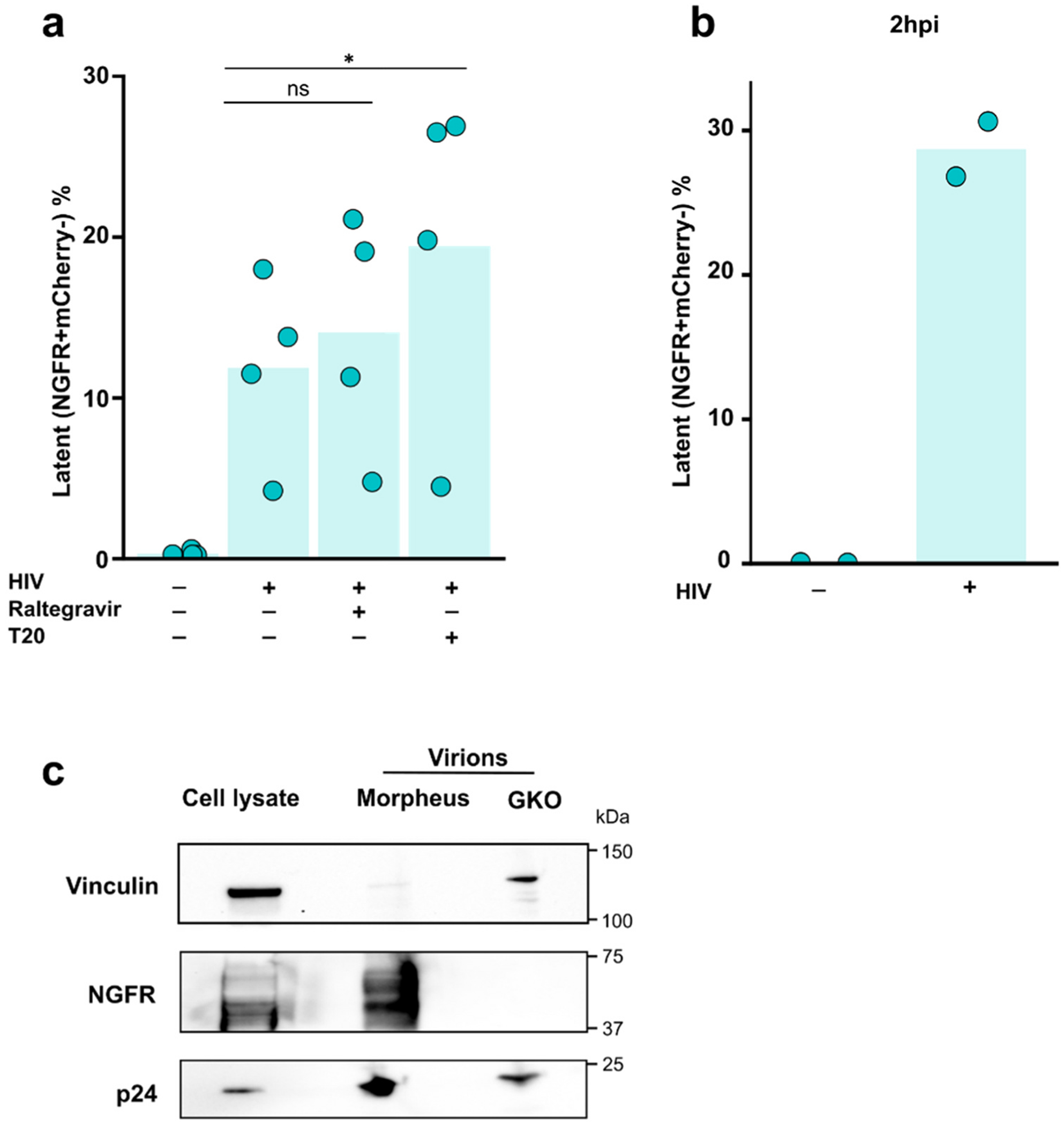
Virion attachment to the cell surface contributes to the percentage of cells detected as NGFR+ . (a) Resting CD4 + T-cells were infected with Morpheus-V5 virus at tissue culture infectivity dose (TCID) 50 per cell of 0.005 in the presence of 1uM Raltegravir or 4.45uM T20 fusion inhibitor and five days post-infection, cells were harvested and the percentage of cells expressing NGFR+mCherry− (latent, blue) in live cells was quantified. The symbols represent individual donors and the height of the column the mean of n = 4 individual donors. (b) Resting CD4 + T-cells were infected with Morpheus-V5 virus at TCID 50 per cell of 0.005 and two hours post infection, cells were harvested and the percentage of cells expressing NGFR (blue) in live cells quantified. The symbols represent individual donors and the height of the column the mean of n = 2 individual donors. (c) HEK293T cells were transfected with pMorpheus-V5 virus or with another dual reporter virus using the GKO plasmid (which encodes GFP as the productive marker and EF1α (Elongation factor 1α) driven-mKO2 (mKusabira-Orange2) as the latent marker) pseudotyped with envelope from NL4.3. Supernatant was collected and concentrated for virus production. Transfected cells and virions were lysed for western blot analysis and NGFR protein expression levels quantified in lysed transfected cells and virions. The dual reporter virus GKO was used as a negative control for NGFR expression. Statistical significance was calculated using a paired *t*-test. Not significant (ns) p > 0.05; *p < 0.05.

We hypothesised that NGFR+mCherry- (latent) cells that didn’t contain HIV DNA was a result of cells with virions either bound on the outside or fused with the cell membrane. To investigate this, CD4 + T-cells were infected with Morpheus-V5 virus in the presence of an integrase inhibitor (raltegravir) or a fusion inhibitor (T20). In the presence of raltegravir, we observed similar levels of NGFR+mCherry- (latent) cells (mean±SEM=11.88 ± 5.77 % without any inhibitors and 14.07 ± 7.50 % with raltegravir, Fig. 3a). Interestingly, the number of NGFR+mCherry- (latent) cells was higher in the presence of T20 (mean ±SEM 19.42 ± 10.47 %, comparison to no inhibitor, p = 0.035, Fig. 3a), consistent with NGFR presence in the absence of viral entry and integration, indicating that the NGFR signal from unintegrated virus was contributing to the estimates of NGFR+mCherry- (latent) cells.

Given the above findings, we hypothesised that the virus stock expressed NGFR protein and as soon as the cells come in contact with the virus, NGFR protein would be detected. Indeed, the NGFR protein was detected on target cells two hours post infection (Fig. 3b). We used western blots to measure NGFR protein and found that it was expressed in 293HEKT cells following transfection with the pMorpheus-V5 plasmid (Fig. 3c) and was also detected in concentrated virus preparations derived from the transfected 293HEKT cells (Fig. 3c), consistent with the latent reporter protein being incorporated in the virion preparations during the virus production step. Taken together, these data demonstrate that the NGFR protein can be incorporated into virion preparations leading to an overestimation of the NGFR+mCherry- (latent) population. The magnitude of the NGFR signal reduced over time in culture, likely as a result of virion decay from the cell surface.

### 3.4. Using CD4 + T-cells infected with Morpheus-V5 to quantify the effects of latency reversing agents

**Fig. 4.**
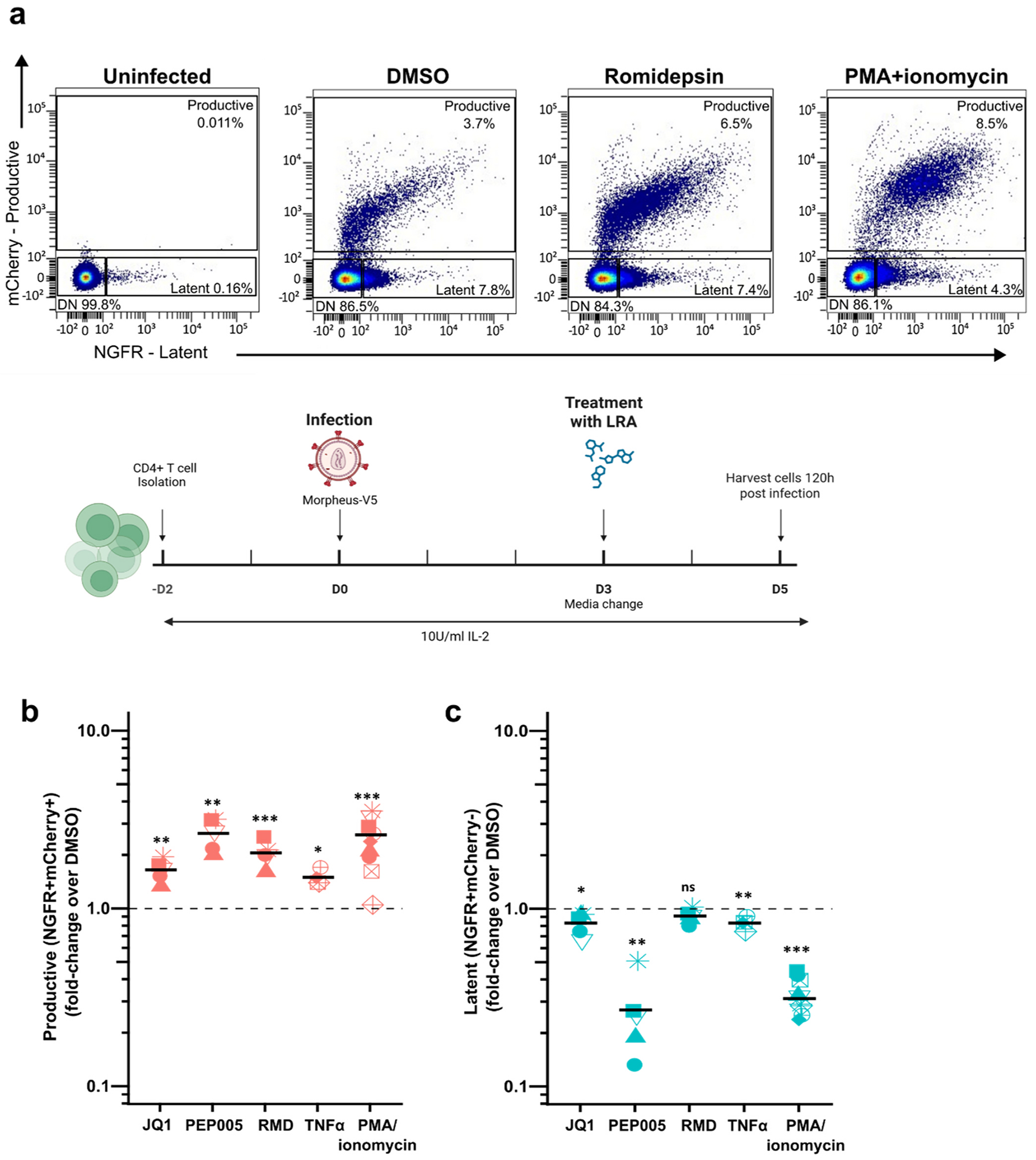
Impact of latency reversal agents on productive and latent populations following infection of CD4 + T-cells with Morpheus-V5. Resting CD4 + T-cells were infected with Morpheus-V5 virus at tissue culture infectivity dose (TCID) 50 per cell of 0.005. Three days post-infection cells were treated with DMSO (negative control), 16 nM Phorbol 12-myristate 13-acetate (PMA), 0.5uM ionomycin (positive control) or latency reversing agents (1uM JQ1, 50 nM PEP005, 20 ng/ml TNFα and 20 nM romidepsin (RMD)) for 48 h, except for romidepsin which was washed out after four hour to reduce toxicity. Expression of mCherry and NGFR were quantified by flow cytometry. (a) Representative flow plots of uninfected and infected cells 48 h following treatment with DMSO (negative control), romidepsin and PMA/ionomycin (positive control) and a schematic representation of the experimental design. Fold-change relative to DMSO of live (b) productive cells (NGFR+mCherry+, red) and (c) latent cells (NGFR+mCherry−, blue) following treatment with the different latency reversing agents. Horizontal line represents the mean of n = 4–5 individual donors for LRAs and n = 12 for PMA/ionomycin. Dashed horizontal line at one represents no change. Statistical significance relative to DMSO treatment was calculated using a paired *t*-test on raw values. Not significant (ns) p > 0.05; *p < 0.05; **p < 0.01; ***p < 0.001.

We sought to determine whether the Morpheus-V5 reporter system could be used as a primary cell model to screen latency reversing agents (LRAs). Resting CD4 + T-cells were infected with the reporter virus and treated with a range of LRAs including JQ1, PEP005, romidepsin, tumour necrosis factor alpha (TNFα) and PMA/ionomycin (positive control) (Fig. 4a). We hypothesised we would observe a reduction in the NGFR+mCherry- (latent) cells with an increase in the NGFR+mCherry+ (productive) cells, indicative of proviral reactivation in NGFR+mCherry- (latent) cells.

Treatment with JQ1, PEP005, romidepsin and TNFα resulted in an increase in NGFR+mCherry+ (productive) cells (mean±SEM fold change of mCherry and NGFR (productive marker) expression compared to DMSO was 1.65 ± 0.11, 2.65 ± 0.24, 2.06 ± 0.15 and 1.5± 0.07; p = 0.002, p = 0.001, p = 0.001 and p = 0.012 respectively, Fig. 4b) with a clear increase with the positive control, PMA/ionomycin, as expected (2.60 ± 0.26; p = 0.001, Fig. 4b). There was also a reduction in the NGFR+mCherry- (latent) population following treatment with the LRAs (Fig. 4c). We observed a significant decrease of NGFR+mCherry- (latent) cells following JQ1, PEP005 and TNFα, (mean±SEM fold change of NGFR expression compared to DMSO was 0.83 ± 0.05, 0.27 ± 0.06, 0.83 ± 0.03; p = 0.041, p = 0.005, p = 0.009 respectively, Fig. 4c). There was no significant decrease in NGFR+mCherry- (latent) cells with romidepsin (0.91 ± 0.04; p = 0.121, Fig. 4c). The positive control, PMA/ionomycin, also induced a marked reduction, as expected (0.31 ± 0.03; p < 0.001, Fig. 4c). The magnitude of the reduction in the NGFR+mCherry- (latent) population with cell activating agents (PEP005 and PMA/ionomycin) was greater than the increase in NGFR+mCherry+ (productive) cells, which could potentially be explained by cellular proliferation diluting the expression of NGFR or cellular activation leading to enhanced endocytic uptake and therefore reduced expression of NGFR (Rossatti et al., 2022).

### 3.5. Using CD4 + T-cells infected with Morpheus-V5 to quantify the effects of pro-apoptotic drugs

**Fig. 5.**
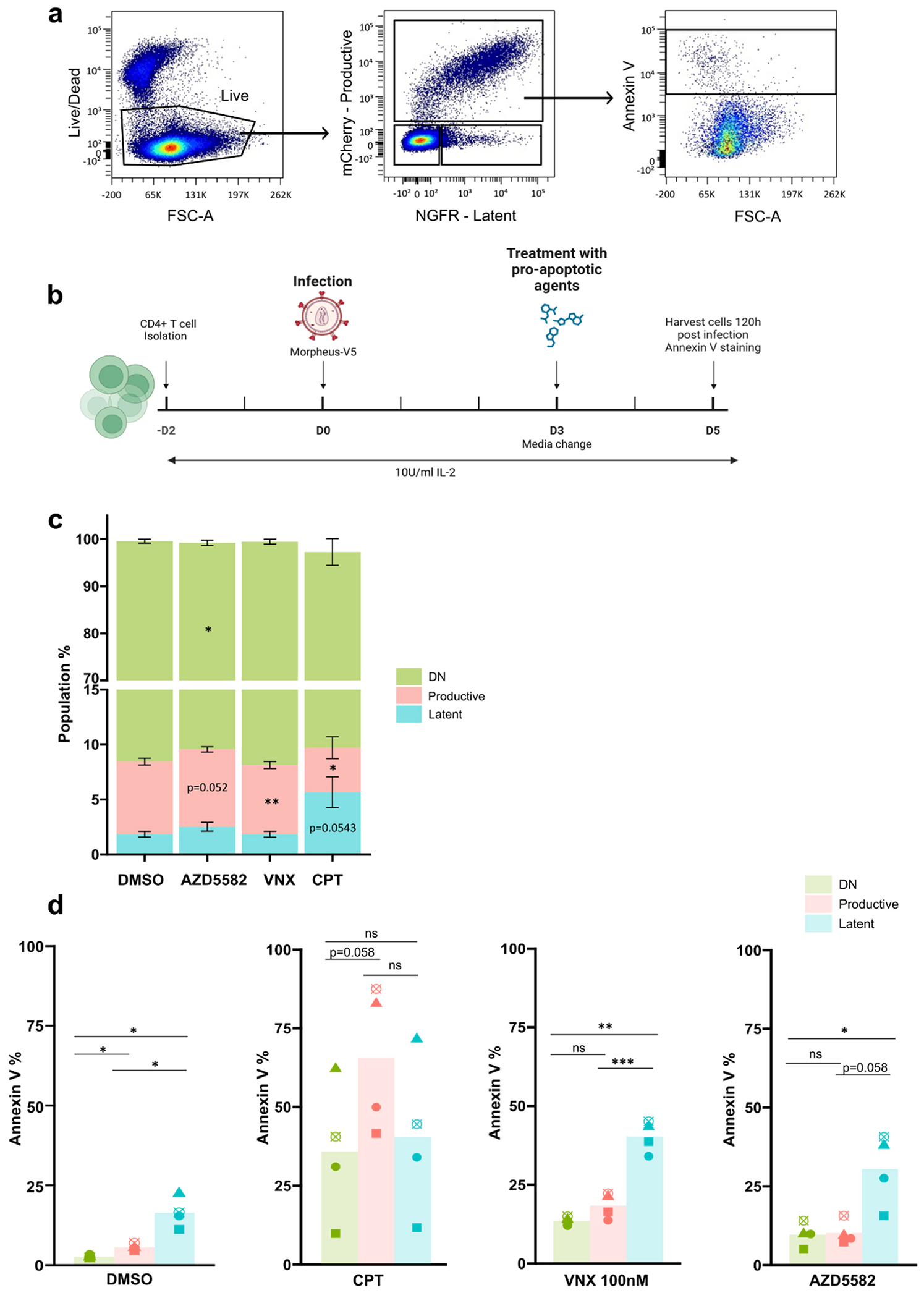
Impact of pro-apoptotic agents on productive and latent populations following infection of CD4+ T-cells with Morpheus-V5. Activated CD4+ T-cells were infected with Morpheus-V5 virus and after 72h, 0.1% DMSO, 5uM camptothecin (CPT), 100 nM venetoclax (VNX) or 100 nM AZD5582 were added. After 48h incubation, cells were harvested and analysed by flow cytometry. (a) Representative flow plots and gating strategy. (b) Schematic representation of the experimental design. (c) The proportion of the total population of surviving cells that were double negative (uninfected, green), NGFR+mCherry+ (productive, red) and NGFR+mCherry− (latent, blue) cells post treatment on day five. The height of the column and error bars represent mean±SEM of n = 4. Statistical significance was calculated using a paired *t*-test. (d) Percentage of double negative (uninfected, green), NGFR+mCherry+ (productive, red) and NGFR+mCherry− (latent, blue) cells that were Annexin V positive after five days in culture with DMSO alone, camptothecin (CPT), venetoclax (VNX) or AZD5582. The symbols represent individual donors and the height of the column the mean of n = 4 individual donors. ANOVA followed by post-hoc pairwise paired t-tests with the Bonferroni multiple testing correction. Not significant (ns) p > 0.05; *p < 0.05; **p < 0.01; ***p < 0.001.

We next determined if infection with Morpheus-V5 virus could provide insights into cell death in productively and latently infected cells following incubation with pro-apoptotic drugs and incorporated annexin V staining, an early apoptosis marker (Fig. 5a, b).

We looked at the effect of pro-apoptotic drugs on the different populations. Camptothecin (CPT), a DNA topoisomerase inhibitor that induces apoptosis (Liu et al., 2000) was used as our positive control. AZD5582 is a SMAC mimetic that can reverse HIV latency and induce apoptosis through inhibition of inhibitors of apoptosis (Nixon et al., 2020; Moon et al., 2015) and venetoclax inhibits B-cell lymphoma-2 and can induce selective death of HIV-infected cells (Nixon et al., 2020; Arandjelovic et al., 2023). Treating cells with AZD5582, venetoclax or camptothecin post infection resulted in changes in the distribution of NGFR+mCherry+ (productive) and NGFR+mCherry- (latent) cells in the surviving population (Fig. 5c). Campothecin compared to DMSO induced a reduction in the frequency of NGFR+mCherry+ (productive) cells by 0.59-fold (p = 0.033, Fig. 5c) accompanied by a 3.02-fold increase in the NGFR+mCherry- (latent) population (p = 0.054, Fig. 5c). Compared to DMSO, venetoclax treatment led to a moderate decline in the NGFR+mCherry+ (productive) population by 0.95-fold (p = 0.008, Fig. 5c). AZD5582 treatment led to a small decline in the double negative (uninfected) population (fold change compared to DMSO was 0.98, p = 0.018, Fig. 5c), which was accompanied by a modest increase in the NGFR+mCherry- (latent) and NGFR+mCherry+ (productive) populations (fold change over DMSO was 1.37 and 1.07; p = 0.067 and p = 0.052 respectively, Fig. 5c).

We then looked at the levels of annexin V staining in cells following treatment with the control and pro-apoptotic drugs. Five days following infection with Morpheus-V5 virus, the annexin V signal was quantified in double negative (uninfected), NGFR+mCherry+ (productive) and NGFR+mCherry- (latent) infected cells in DMSO treated cells (mean+SEM=2.91 ± 0.24 %, 5.84 ± 0.50 % and 16.58 ± 2.31 % respectively, Fig. 5d). Surprisingly, NGFR+mCherry- (latent) cells showed higher annexin V staining than NGFR+mCherry+ (productive) or double negative (uninfected) cells (p = 0.048 and p = 0.029 respectively, Fig. 5d). To confirm that this wasn’t due to non-specific binding of anti-NGFR antibody to dying cells (and therefore falsely classifying apoptotic cells as latently infected), we quantified the NGFR expression of dead (annexin V+) and live (annexin V−) populations in the absence of virus infection by first gating on live (live/dead-) cells followed by dead (annexin V+) or live (annexin V−) cells (Fig S3a). We saw no NGFR expression in the cells that were either live or dead (Fig S3b). These data indicate that apoptotic cells are unlikely to be incorrectly labelled as NGFR+mCherry- (latent) and that high annexin V signal observed in the latent population is due to true signal and not due to non-specific binding of NGFR antibody to dying cells (Fig S3b).

The positive control campothecin increased annexin V staining in double negative (uninfected), NGFR+mCherry+ (productive) and NGFR+mCherry- (latent) cells (35.85 ± 10.85 %, 65.45 ± 11.54 %, 40.43 ± 12.41 % respectively, Fig. 5d), with the highest levels of annexin V staining in the NGFR+mCherry+ (productive) cells (comparison of NGFR+mCherry+ (productive) and double negative (uninfected) cells, p = 0.058, Fig. 5d). Following treatment with venetoclax and AZD5582, the staining levels of annexin V was highest in the NGFR+mCherry- (latent) cells (40.03 ± 2.48 % and 30.70 ± 5.68 % respectively; comparison to double negative (uninfected) cells, p = 0.002 and p = 0.044 respectively, Fig. 5d). Following venetoclax treatment, the level of annexin V was higher in NGFR+mCherry- (latent) compared to NGFR+mCherry+ (productive) cells (p < 0.001, Fig. 5d) with similar trends following AZD5582 treatment, although these differences did not reach statistical significance (p = 0.058, Fig. 5d). Together these data highlight that the relative susceptibility of latently and productively infected cells to apoptosis can be quantified using drugs that activate different apoptosis pathways.

## 4. Discussion

Here we evaluated the Morpheus-V5 dual reporter virus and showed that Morpheus-V5 could be used to understand interventions that target latency, but the virus had some limitations. We observed that Morpheus-V5 overestimated the number of NGFR+mCherry- (latent) cells, likely as a consequence of NGFR incorporation into virions and non-specific adherence of virions to uninfected cells. Nevertheless, NGFR+mCherry- (latent) cells were still enriched for infection, although very few NGFR+mCherry- (latent) cells had inducible virus following a single round of infection. We demonstrated that latency reversal could be quantified using this model as both a reduction in NGFR+mCherry- (latent) cells and an increase in NGFR+mCherry+ (productive) cells. Finally, we observed differential susceptibility to death in double negative (uninfected), NGFR+mCherry+ (productive) or NGFR+mCherry- (latent) cells following incubation with pro-apoptotic compounds.

We demonstrated that a membrane bound protein as a marker for latent HIV infection may lead to an overestimation of the frequency of latent cells. Despite these findings, expression of NGFR did enrich for cells containing HIV DNA and also inducible virus. Furthermore, we demonstrated that the enrichment improved with more prolonged in vitro culture, however, even after 120 days, only 5% of NGFR+mCherry− (latent) cells contained inducible virus. We propose several explanations for NGFR+mCherry− cells that were not infected with HIV DNA. First, NGFR+mCherry− cells that did not contain HIV DNA could have arisen from incorporation of NGFR protein into virions (as we show here) or microvesicles (Bess et al., 1997). Another possible explanation of NGFR+mCherry− (latent) cells without HIV DNA could be abortive infection where reverse transcription and therefore integration was not complete. This could potentially also explain the high basal level of apoptosis in the NGFR+mCherry− cells following infection of resting but not activated cells, and prior reports of abortive infection in resting T cells (Doitsh et al., 2010). Interestingly, significant abortive infection has been observed in another HIV reporter virus system that measured both virion fusion and productive infection (Tilton et al., 2014). Finally, given the findings that T20 increased the percentage of NGFR+mCherry− (latent) cells, it is possible that T20 interfered with NGFR recycling at the cell surface.

We also showed that the Morpheus-V5 virus could be used to study apoptosis in the setting of HIV infection and that latently and productively infected cells had varying susceptibility to death following treatment with different proapoptotic drugs. The drugs we tested differed by their mechanisms of action; CPT induces apoptosis via damaging DNA (Liu et al., 2000), AZD5582 inhibits inhibitors of apoptosis proteins and induces TNFα receptor mediated extrinsic apoptosis (Hennessy et al., 2013; Fotin-Mleczek et al., 2002), whereas venetoclax is a BH3-mimetic that inhibits the prosurvival BCL-2 protein and induces the intrinsic apoptosis pathway (Souers et al., 2013). Such differences may explain the different patterns of apoptosis observed in this study. Understanding which cells were more or less susceptible to pro-apoptotic drugs could provide important insights for future use of specific interventions. For example, our team recently showed that venetoclax reduced the frequency of cells carrying intact HIV DNA in *ex vivo* CD4+ T-cells from people with HIV (PWH) on ART (Arandjelovic et al., 2023) but were unable to determine in this assay if the decline was due to a loss of latently or productively infected cells. Findings using the Morpheus-V5 virus in our study were consistent with venetoclax enhancing death of latently infected cells.

Our study had some limitations. First, we were unable to determine whether NGFR+mCherry− (latent) cells in the Morpheus-V5 were a result of direct infection of resting cells (pre-activation latency) or the transition of productive infection to latent infection as a result of changes in cell activation state (post activation latency) (Shan et al., 2017). Second, we were unable to differentiate between intact and defective viruses in the latently infected cells, and others have shown that up to 40% of proviruses are defective after one round of in vitro infection (Bruner et al., 2016). However, we demonstrated that expression of NGFR enriched for cells with inducible virus. Third, although we demonstrated the presence of NGFR protein in virion preparations, we didn’t conclusively show that NGFR was incorporated into the virions. Fourth, we only quantified one marker of apoptosis, Annexin V which is an early marker, and therefore may have under estimated the number of dying cells. Finally, we acknowledge that this *in vitro* model doesn’t fully reflect the biology of latently infected cells *in vivo*, which are subject to immune pressure and selection over time.

## 5. Conclusions

The Morpheus-V5 dual reporter virus is a valuable tool for studying latency as well as interventions to disrupt latency, including latency reversing agents and pro-apoptotic drugs and can provide insights into the impact of these interventions in both latently and productively infected cells. It was possible to pseudotype Morpheus-V5 with diverse envelopes, allowing for study of latency in CD4+ T-cells and monocyte-derived macrophages. There are some limitations of the dual reporter virus in that expression of the reporter only modestly enriched for latently infected cells, meaning that expression of NGFR over estimated the true frequency of latently infected cells.

## Supplementary Material

1

## Data Availability

Data will be made available on request.

## Appendix A. Supporting information

Supplementary data associated with this article can be found in the online version at doi:10.1016/j.jviromet.2025.115236.
