# Supplementary material for: Limitations and use of the Morpheus-V5 dual reporter virus in assessing interventions that target HIV latency
Source: J Virol Methods. Author manuscript; Available in PMC 2026 Jun 21. (PMC13283291; doi:10.1016/j.jviromet.2025.115236)
Supplement: 1 [file NIHMS2180475-supplement-1.docx]

**Supplementary data**


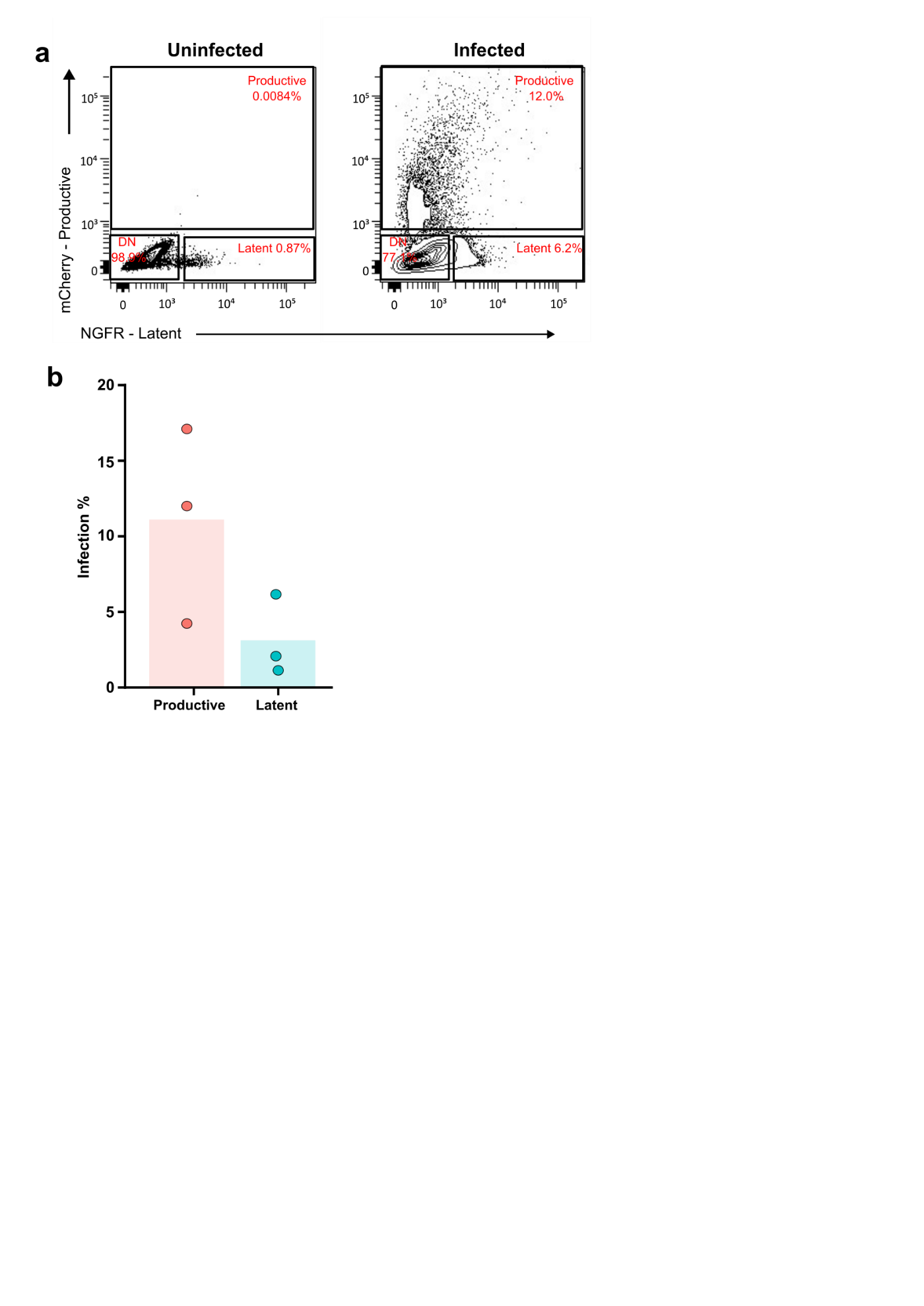


**Supplementary Figure 1: Latent infection can be established in monocyte-derived macrophages infected with Morpheus-V5 virus.** Monocyte-derived macrophages were infected with YU2 pseudotyped Morpheus-V5 virus and five days post infection, cells were harvested. (a) Representative flow plots of uninfected and infected macrophages. (b) The percentage of NGFR+mCherry+ (productive, red) and NGFR+mCherry- (latent, blue) cells. The symbols represent individual donors and the height of the column the mean of n=3 individual donors.


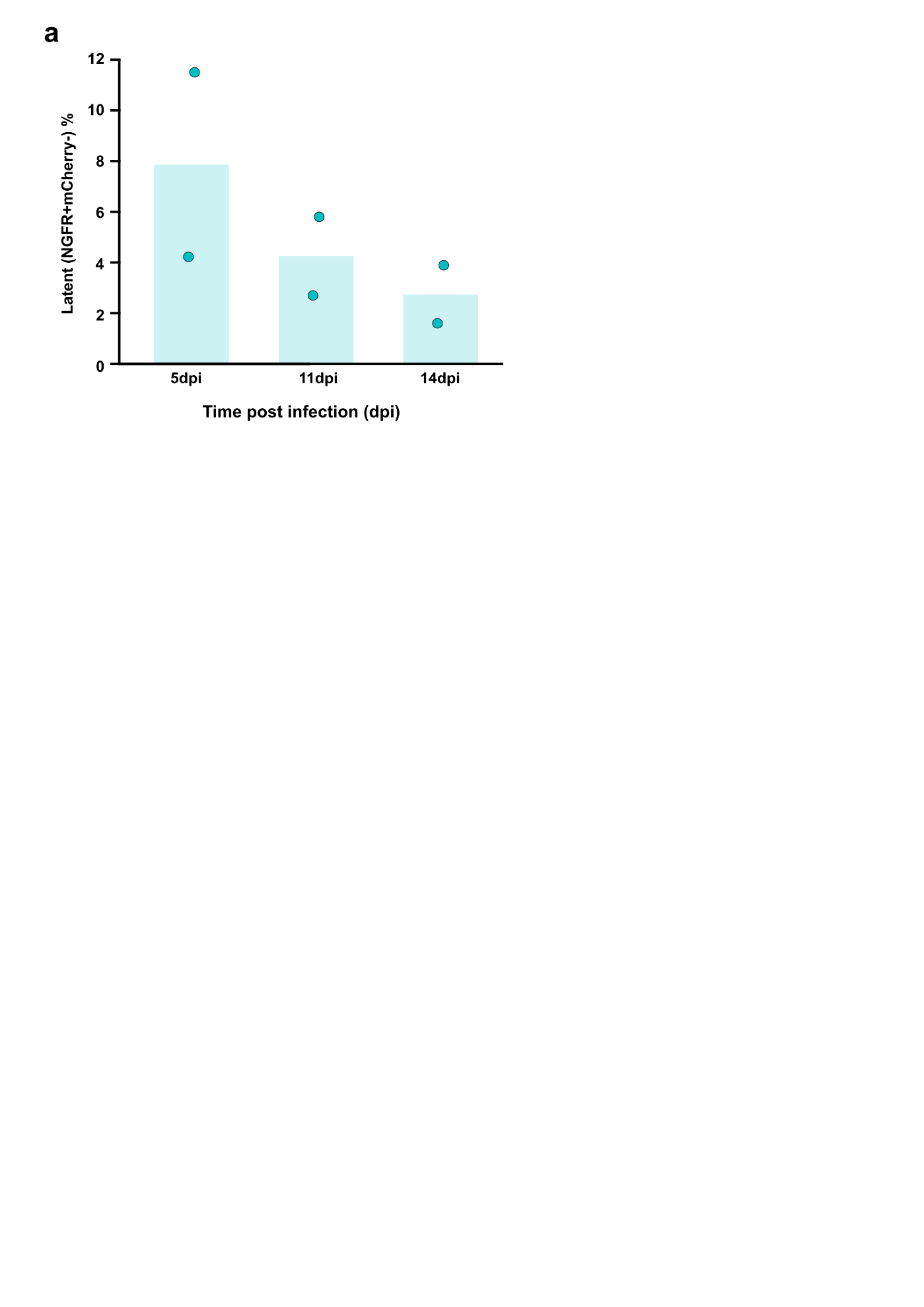


**Supplementary Figure 2: Reduction in the number of latently infected cells following long-term culture of CD4+ T-cells infected with Morpheus-V5 virus.** Resting CD4+ T-cells were infected with Morpheus-V5 virus at a tissue culture infectivity dose (TCID) TCID 50 per cell of 0.005 and harvested after 5, 11 and 14 days post infection (dpi). A media change was performed on day 5, 8 and 11. The percentage of NGFR+mCherry- (latent) cells at various timepoints. The symbols represent individual donors and the height of the column the mean of n=2 individual donors.


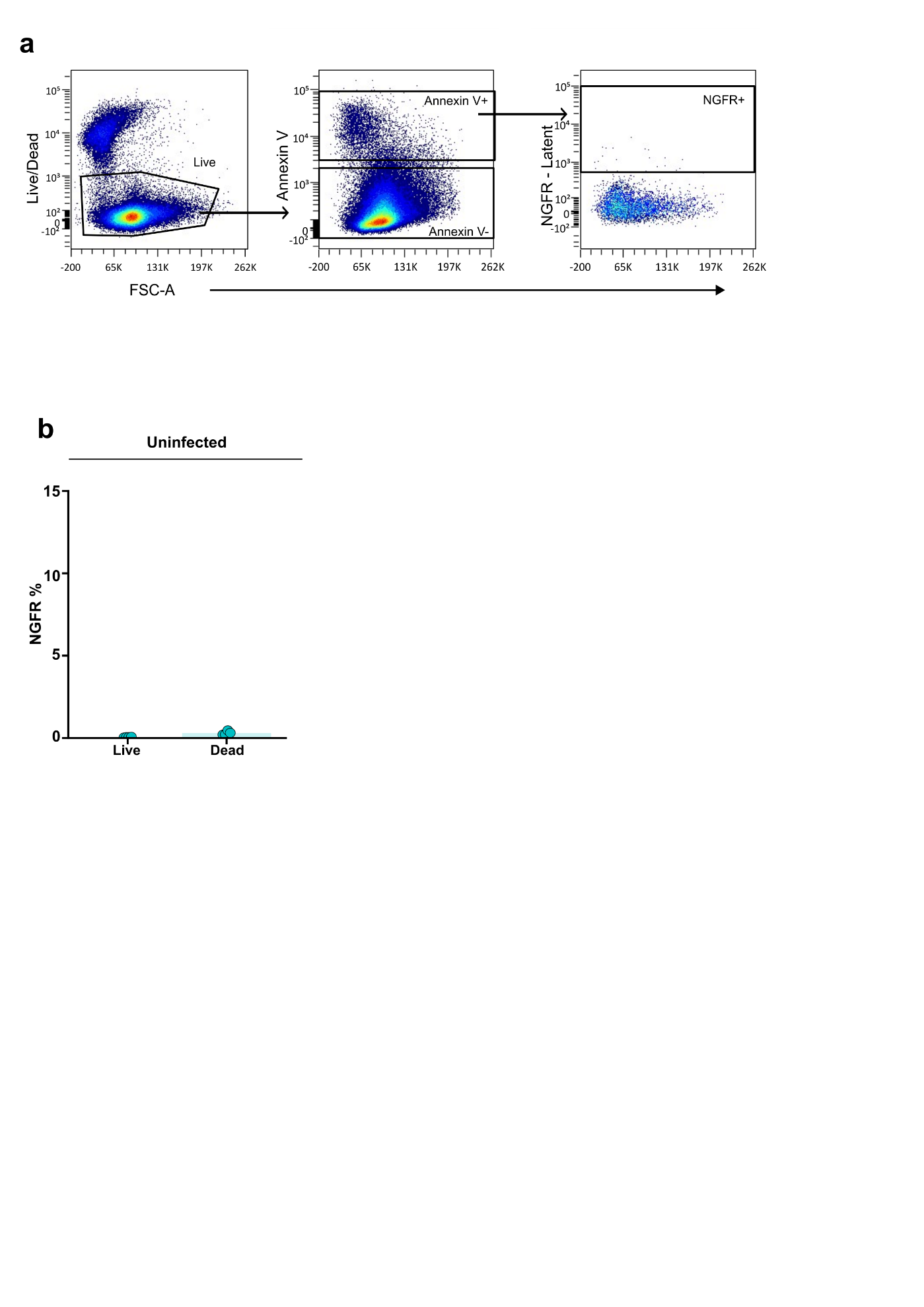


**Supplementary Figure 3: Proapoptotic compounds did not lead to expression of NGFR on dead cells.** Activated and mock infected CD4+ T-cells were stained with NGFR antibody and analysed by flow cytometry on day five. (a) Representative flow plots and gating strategy. Cells were first gated on live (live/dead-) cells followed by live (annexin-) and dead (annexin V+) cells. (b) The percentage of NGFR+ cells (blue) in live (annexin V-) and dead (annexin V+) cells. The symbols represent individual donors and the height of the column the mean of n=4 individual donors.
